# Rack‐Integrated Quantum Dot‐Based Source of Single and Entangled Photons at Telecom C‐Band

**DOI:** 10.1002/nap2.70353

**Published:** 2026-11-03

**Authors:** Michal Vyvlecka, Raphael Joos, Benjamin Breiholz, Emma Marmasse, Anna Friederike Köhler, Ponraj Vijayan, Tobias Huber‐Loyola, Sven Höfling, Michael Jetter, Simone Luca Portalupi, Peter Michler

**Affiliations:** ^1^ Institut für Halbleiteroptik und Funktionelle Grenzflächen Center for Integrated Quantum Science and Technology (IQ^ST^) and SCoPE University of Stuttgart Stuttgart Germany; ^2^ Julius‐Maximilians‐Universität Würzburg Physikalisches Institut and Würzburg‐Dresden Cluster of Excellence ctd.qmat Würzburg Bavaria Germany; ^3^ Institute of Photonics and Quantum Electronics (IPQ) and Center for Integrated Quantum Science and Technology (IQST) Karlsruhe Institute of Technology (KIT) Karlsruhe Germany

**Keywords:** deployed quantum communication, quantum dots, telecom C‐band

## Abstract

Bringing quantum light sources into real‐world telecommunication networks requires moving beyond the laboratory and developing sources that operate at the telecom C‐band. Epitaxial quantum dots (QDs) provide deterministic sources of quantum light at these wavelengths. Here, we demonstrate an important step toward quantum communication networks: a state‐of‐the‐art semiconductor QD source, exhibiting a record coincidence rate for entangled‐photon emission in the telecom C‐band, integrated into an optimised rack‐based setup exhibiting remote controle. This setup includes a tunable pulsed laser for QD excitation, enabling operation from quasi‐resonant to resonant two‐photon excitation, all‐optical excitation suppression, and QD signal coupling into single‐mode fibres. Overall, the setup achieves more than 65% transmission for both exciton and biexciton photons. To demonstrate the usability of our stand‐alone source, we transmit single photons through a deployed 36 km‐long fibre link and verify that their single‐photon purity is preserved. Moreover, based on the measured entangled‐photon pair rate, we estimate that the source could enable entangled QKD protocols with a projected secret key rate 3.4 cps after 150 km of optical fibres. These results demonstrate the potential of quantum‐dot‐based telecom light sources for integration into existing fibre infrastructures, paving the way toward the realisation of a quantum internet.

## Graphical abstract

A portable, rack‐integrated quantum dot‐based single and entangled photon source enables stable long‐term operation and remote control at deployed locations. An optimised optical setup provides record coincidence rates of high‐fidelity entangled‐photon pairs generated in the telecom C‐band. The system offers a practical platform for entanglement‐based quantum communication over deployed fiber networks.

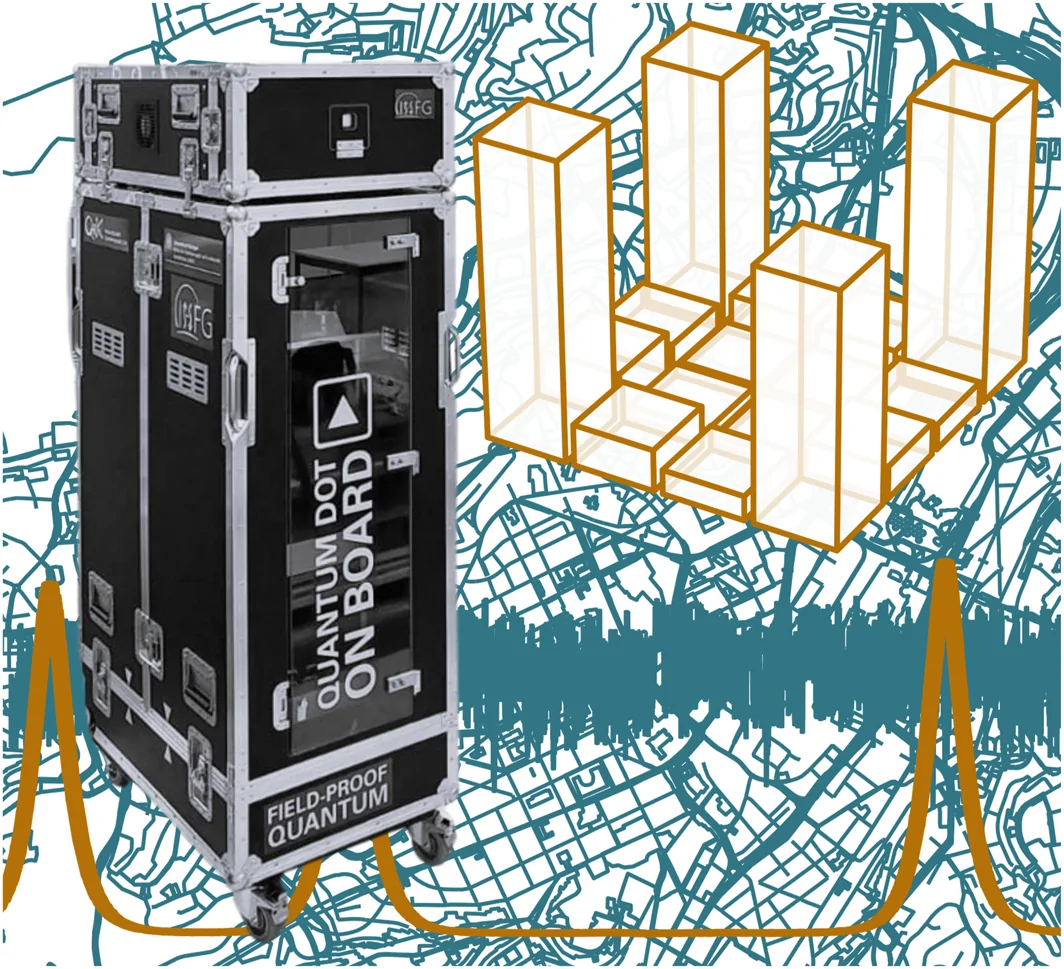

## 1 Introduction

The World Wide Web is currently the heart of our global communication, having various distant nodes connected via silica fibres and over satellite links. Quantum technologies are expected to affect everyday life, especially secure communication, quantum sensing, and access to remote quantum computers. This expectation has motivated efforts to move quantum devices from laboratory demonstrations toward practical technologies. [1]. This requires an accurate design optimisation of each building block, particularly drastically reducing the overall footprint, ensuring remote control of the setups, and enabling high stability in environments where mechanical vibration and temperature instabilities may be larger than in well‐controlled quantum optics laboratory.

When employing quantum light for communication schemes, the two main building blocks will be the source itself, and the receiver station. For quantum light generation, epitaxial quantum dots are raising interest as per their state‐of‐the‐art emission properties as well as for the deterministic nature of the generation process [2]. Although interesting experiments have been conducted under electrical pumping [3, 4, 5], optical excitation still provides the highest performance of the emitted photons in terms of single‐photon source purity and photon coherence [6, 7, 8]. This translates in the need for wavelength‐tunable, pulsed mode‐locked lasers in all laboratory experiments.

The main limitation of the deployed implementation of QDs stems from the necessity of operating them at cryogenic temperatures (around 4 K) in a thermally‐stable environment. To partially overcome this limitation, QDs were tested at higher operation temperatures over 15 K, allowing their integration in a more compact and affordable Stirling cooler‐based cryostats [9, 10]. However, higher operation temperatures strongly influence the single‐photon source purity, which is the key parameter for long‐distance quantum cryptography, and it also influences other parameters such as photon indistinguishability [11, 12], which is crucial for fundamental quantum operations in photonic quantum repeaters. This motivated the realisation of smaller rack‐based setups, which demonstrated operation of QDs emitting at wavelengths around 780 nm [13].

Here we present a compact, transportable, and rack‐based setup that constitutes a source of single‐ and polarisation‐entangled photon pairs at the telecom C‐band. This is enabled by the combined use of a telecom InAs/GaAs quantum dot, further operated at 4K to ensure the highest optical performances, with a compact setup including optics control and analysis. Indeed, to be useful in deployed scenarios, the source unit comprises all necessary elements for a fully stand‐alone operation of the source, that is a compact cryostat, a pulsed laser, optical elements for the photon collection and laser filtering, and remotely controlled optomechanics. From the perspective of the user, the telecom photons are directly available at the output of a single‐mode fibre (respectively, two distinct single‐mode fibres) for the operation as source of single‐photons (source of polarisation‐entangled photon pairs) in the telecom C‐band. In addition, a second rack is built as a receiver unit, containing state‐of‐the‐art superconducting nanowire single‐photon detectors (SNSPDs) and all‐optical elements for conducting quantum optical measurements: these span from the estimation of the single‐photon nature of the emission (Hanbury‐Brown and Twiss setup), quantification of the degree of entanglement (via quantum state tomography) to a quantum cryptographic receiving unit. In the following paper, we will highlight in detail the setup configuration, showing operation as source of single photons and entangled photon pairs, and their use in a field‐deployed fibre line across the University of Stuttgart‐campus Vaihingen, and the city of Stuttgart, reaching almost 36 km with 18 dB of loss.

## 2 Portable Quantum‐Dot Single‐Photon Source

The portable quantum‐dot single‐photon source was designed to address the following requirements and challenges associated with the deployment of quantum light sources outside a controlled laboratory environment: (i) The setup is fully enclosed within a transportable box, which isolates the system from environmental fluctuations, such as mechanical vibrations, stray light, and temperature fluctuations, and allows the system to be readily transported using standard transportation facilities. (ii) Potential variations in excitation power and polarisation transformations in the deployed fibre link are compensated using automatic protocols. (iii) The collection efficiency of the emitted photons is maximised by minimising the optical path length and using optimised optical elements, such as high‐reflectivity and low‐group‐delay‐dispersion (GDD) mirrors. (iv) The system is suitable for deployed operation owing to the robust design of the optical setup, which relies mostly on passive optical elements, such as beamsplitters, dichroic mirrors, and volume Bragg gratings (VBGs), complemented by remotely controllable active elements, including rotating waveplates, MEMS switches, and flipping mirrors. In the following, we describe the physical implementation and optical design of the portable system in more detail.

The central component in the portable setup is the compact closed‐cycle optical cryostat (attoDRY800xs), pumped by an air‐cooled helium compressor. This configuration allows the quantum dot single‐ and entangled‐photon source to operate at a temperature of 4K without requiring special infrastructure, such as access to cooling water. The optical setup for quantum dot excitation and single‐photon collection is divided into three floors: the excitation floor, the collection floor, and the cryostat floor. All three are mounted on independent breadboards positioned above the cryostat vacuum chamber and connected to the cryostat via vibration‐damping posts.

Importantly, all active optical elements are remotely controllable, enabling independent operation of the system at a deployed location, without requiring the user to be physically present at the experimental site. The main elements of the system can be operated and monitored remotely using dedicated control software and remote‐access capabilities. These include the cryostat cooldown, excitation‐laser power and polarisation control, polarisation‐tomography measurements, polarisation compensation of the deployed fiber, adjustment of the volume Bragg gratings for spectral filtering, and switching between the characterisation and main measurement ports. Thus, although the system still requires an initial optical alignment, its main operating functions do not require manual adjustment of individual optical components.

The entire system is enclosed in a custom‐built transportable box with dimensions of 600mm×1649mm×1003mm (W × H × D), mounted on four heavy‐duty wheels with a radius of 125 mm. The transportable box is equipped with all necessary connections for operating the system in a deployed scenario while fully closed. The control electronics, together with the pulsed fibre excitation laser, are housed in a separate box containing two 19 racks with a total height of 10 standard rack units. When the setup transport box and the electronics box are stacked, the total height of the system is 1980 mm, making it compatible with transport through all standard facilities.

### 2.1 Excitation Floor

Key to the operation of a quantum light source based on epitaxial quantum dots is the optical excitation method. In this setup, the quantum dot can be excited by three independent light sources, which are combined using a dichroic mirror into a single spatial mode within the excitation floor (see Figure 1, green block). (i) The first source is a collimated white‐light source controlled by an external trigger. It is used to image the sample surface and to provide additional above‐band‐gap illumination, which stabilises the free‐carrier environment within the sample [14, 15]. As shown in Ref. [16], the intensity of the two‐colour illumination can affect both the blinking behaviour and the excitation coherence of droplet‐etched GaAs QDs. (ii) The second is a compact continuous‐wave (CW), fibre‐coupled 640 nm laser diode which is employed for above‐band excitation of the quantum dot. Its output power is controlled using a fibre‐coupled MEMS‐based variable optical attenuator. The above‐band laser beam is combined with the white‐light beam into a single spatial mode using a 50:50 beam splitter. (iii) The third is the primary excitation source, which is a pulsed Er‐doped fibre laser with an additional EDFA that provides spectral tunability over the wavelength range from 1530 to 1560 nm; this laser enables the use of various excitation schemes, such as longitudinal acoustic (LA) phonon‐assisted excitation or resonant two‐photon excitation (TPE). It generates 10 ps pulses with a spectral full width at half maximum (FWHM) of 400 pm and a repetition rate that can be tuned stepwise from 76 MHz up to 1 GHz. Spectrally broad background emission originating from the laser fibre amplifier is removed using a fibre‐coupled, narrowband optical filter. The laser power is adjusted with a rotatable half‐wave plate placed between two aligned polarisers, and it is continuously monitored with a power metre.

**FIGURE 1:**
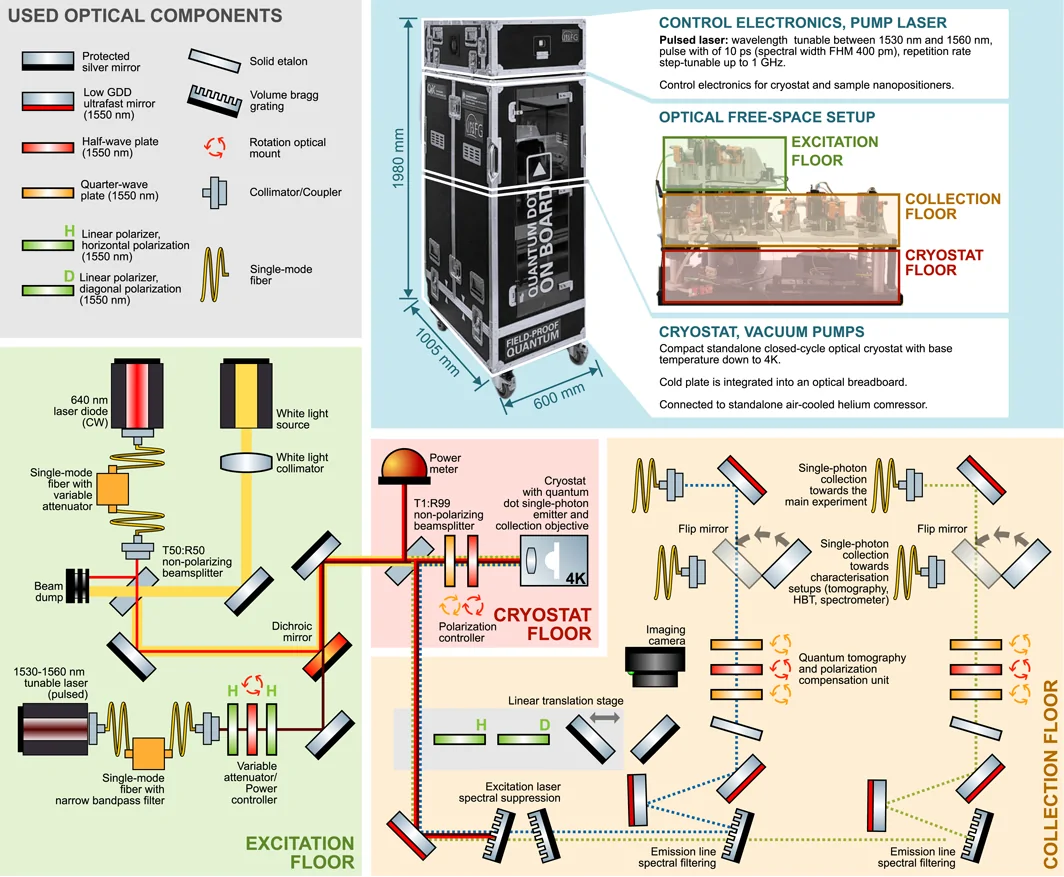
Image and sketch of the transportable source. In the blue block, a picture of the overall setup with a description of each part. The three floors in the optical setup are colour‐coded with respect to the sketch below: green for the excitation floor, orange for the collection floor, and red for the cryostat floor. All light sources and optical and optomechanical elements are visualised. In the grey block, a legend of the used optical elements is shown.

As the combination of the three excitation sources relies exclusively on passive optical components, each source can be controlled independently, while all three can simultaneously illuminate the quantum‐dot sample. This capability is crucial for maintaining a stable charge‐carrier environment during two‐photon excitation measurements.

### 2.2 Cryostat Floor

The excitation light from the above floor is coupled into the cryostat chamber (see Figure 1, red block) through a custom‐made non‐polarising beam splitter with a splitting ratio of 98.5:1.5. This is used to guide 1.5% of the excitation to the sample, while the majority of this light is reflected by the beam splitter toward a power metre, which forms part of the active excitation‐power stabilisation system. The rear surface of the beam splitter is coated 1050nm−1700nm (C‐coating) to suppress back reflections within the optical setup, while the splitting ratio exhibits extremely low polarisation dependence, making it suitable for the efficient collection of polarisation‐entangled photon pairs emitted by the quantum dot. The polarisation state of the excitation beam is controlled using motorised quarter‐wave and half‐wave plates mounted on automatic rotation stages. The beam is then focused onto the sample by a C‐coated lens with a focal length of 3 mm, placed inside the cryostat chamber. The same lens also serves as the collection optic for the photons emitted by the quantum dot. The 98.5% reflection ensures the vast majority of the emitted quantum light is sent to the collection floor.

### 2.3 Collection Floor

The single photons emitted by the quantum dot are separated from the residual excitation laser by a sequence of spectral filters. The first two polarisation‐independent volume Bragg gratings (VBGs) of 1.2nm bandwidth suppress the excitation laser with a suppression ratio of $10^{6}$ per filter while transmitting the emitted light (see Figure 1, orange block): this can be done spectrally as in LA‐phonon assisted pumping and in TPE, emitted light and excitation laser lay at different wavelengths. The two subsequent polarisation‐independent VBGs of 0.55nm bandwidth spectrally separate photons emitted by different quantum‐dot transitions, such as the exciton and biexciton transitions, into two distinct spatial modes.

Each photon is further spectrally filtered by a narrow‐band angle‐tunable optical etalon (Free Spectral Range: 291.6GHz, Finesse: 18.1), providing additional background suppression and improved spectral selectivity. The photons subsequently pass through a sequence of a quarter‐wave plate, a half‐wave plate, and a second quarter‐wave plate, each mounted on an independently controlled motorised rotation stage. This arrangement enables arbitrary single‐qubit polarisation transformations and is used both for quantum state tomography and for polarisation compensation in deployed‐fibre experiments. For the polarisation compensation protocol, the polarisation transformation introduced by the deployed optical fibre is characterised using known input polarisation states, namely horizontal and diagonal polarisation. These states are prepared by calibrated linear polarisers mounted on an automated linear translation stage at the beginning of the collection floor.

Following polarisation control, the photons are either coupled directly into single‐mode fibres for the desired experiment or redirected by a motorised flip mirror into a characterisation arm. The characterisation setups include a Hanbury‐Brown and Twiss setup for second‐order correlation measurements, and polarisation‐analysis modules for quantum state tomography or the photons can be coupled into an external spectrometer. These characterisation instruments are interconnected using a polarisation‐independent MEMS fibre switch, allowing flexible and remote‐controllable routing of the collected photons.

The entire collection setup is designed to maximise the single‐mode fibre coupling and overall transmission efficiency. This is achieved by maintaining a compact optical layout and employing high‐reflectivity and low‐group‐delay‐dispersion (GDD) mirrors throughout the collection path. The low‐GDD mirrors are particularly important for preserving the fidelity of polarisation‐entangled photon pairs, as they minimise uncontrolled polarisation‐dependent phase shifts introduced upon reflection. In addition, the collection floor incorporates a CCD camera for sample imaging and optical alignment. The beam is directed to the camera by a mirror mounted on an automated linear translation stage.

## 3 Optical and Quantum Optical Measurements

In order to demonstrate state‐of‐the‐art operation of the transportable setup as a source of single‐ and polarisation‐entangled photon pairs in the telecom C‐band, InAs/GaAs quantum dots have been used as sources of quantum light. Thanks to a metamorphic buffer layer, the strain is engineered to enable emission in the telecom C‐band [17]. To increase the source brightness, QDs have been realised within a planar λ‐cavity structure, which consists of two Distributed Bragg Reflector (DBR) stacks: a bottom AlAs/GaAs DBR, and a top dielectric DBR composed of alternating ${\text{SiO}}_{2}/{\text{TiO}}_{2}$ layers, as schematically shown in the inset of Figure 2a.

**FIGURE 2:**
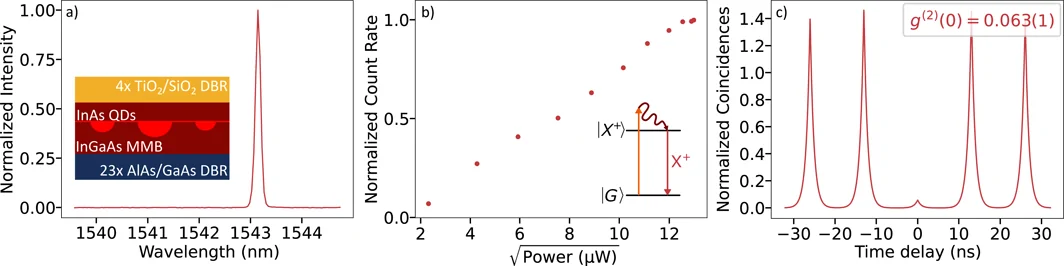
(a) Spectrum of one selected quantum dot under LA‐phonon‐assisted excitation. As the laser is fully suppressed by the spectral filtering, only the QD trion transition $X^{+}$ is visible. In the inset, a schematic of the planar cavity sample, with a bottom semiconductor DBR (23 pairs) and a top oxide DBR (4 pairs). InAs QDs are grown on an InGaAs metamorphic buffer layer (MMB) for strain engineering, enabling emission in the telecom bands. (b) Intensity over excitation power showing a saturation behaviour typical of incoherent excitation methods. In the inset, a schematic energy diagram of the LA‐phonon‐assisted process followed by phonon and photon emission. (c) Normalised photon autocorrelation measurement, $g^{\left(2\right)}\left(\tau \right)$.

### 3.1 Single‐Photon Emission

The optical setup was first characterised using a quantum dot operated under LA‐phonon‐assisted excitation [18]. This excitation scheme enables efficient spectral suppression of the excitation laser while providing high robustness against excitation‐laser power fluctuations and a low multi‐photon emission probability [16, 18, 19]. These properties are particularly important for the implementation of quantum‐dot single‐photon sources in deployed quantum communication and quantum cryptography protocols [20].

A quantum‐dot transition at a wavelength of 1543nm, further referred to as QD‐LA, was excited by a laser spectrally blue detuned by 1.5nm from the transition (see inset of Figure 2b), and operated at a repetition rate of 76MHz. The emission spectrum of QD‐LA after the full suppression of the excitation laser is shown in Figure 2a.

The measured dependence of the single‐photon count rate on the excitation power exhibits the characteristic behaviour of LA phonon‐assisted excitation, reaching a saturation plateau at higher excitation powers (Figure 2b). The normalised single‐photon count rate was extracted from the second‐order correlation measurements. At saturation, a count rate of 1.3 Mcps was measured in the single‐mode fibre‐coupled main collection port, corresponding to an overall end‐to‐end system efficiency of approximately 2%.

The single‐photon nature of the emission was verified by measuring the second‐order correlation function, $g^{\left(2\right)}\left(\tau \right)$. At saturation, the background‐corrected zero‐delay value was measured to be $g^{\left(2\right)}\left(0\right)=0.063\left(1\right)$, confirming strong suppression of residual excitation laser (see Figure 2c). This value can be further reduced by optimising the excitation conditions, particularly the spectral detuning and excitation power [19, 20, 21]. For example, at an excitation power corresponding to 75% of the saturation power, a value of $g^{\left(2\right)}\left(0\right)=0.031\left(2\right)$ was obtained, demonstrating an even lower multi‐photon probability at the expense of a single‐photon count rate reduced to 86% of its saturation value. The QD‐LA's emission shows pronounced blinking; see the Supporting Information. Due to this blinking, the QD‐LA was active for 60.9 (1) % of the time.

### 3.2 Entangled‐Photon Pair Emission

A second QD located on a second chip of a similar design (where instead the bottom DBR is oxide‐based, while the top semiconductor DBR has lower reflectivity) [22] and, hereafter referred to as QD‐TPE, was investigated under TPE. This excitation scheme enables the generation of polarisation‐entangled photon pairs through the biexciton‐exciton radiative cascade [23]. Such entangled photon pairs represent a fundamental resource for entanglement‐based quantum cryptography protocols as well as for entanglement swapping, which is a key building block of photonic quantum repeaters. Furthermore, resonant TPE leads to emission of photons with an ultra‐low multi‐photon contribution [24], which is essential for secure quantum communication, particularly for long communication distances and in mistrustful quantum cryptographic protocols [25].

The exciton and biexciton emission lines are centred at wavelengths of $\lambda_{X}=1532.1\;nm$ and $\lambda_{\text{XX}}=1537.1\;nm$ with respective linewidths $\Gamma_{\text{FWHM,}\;X}=6.93\left(12\right)\;GHz$ and $\Gamma_{\text{FWHM,}\;\text{XX}}=4.27\left(3\right)\;GHz$ [22]. The emission spectrum after suppression of the excitation laser is shown in Figure 3a. During TPE, the free‐charge‐carrier environment of the sample was stabilised by weak additional white‐light illumination. The white‐light intensity was optimised to minimise emitter blinking and thereby maximise the detected photon count rate, as detailed in the Supporting Information.

**FIGURE 3:**
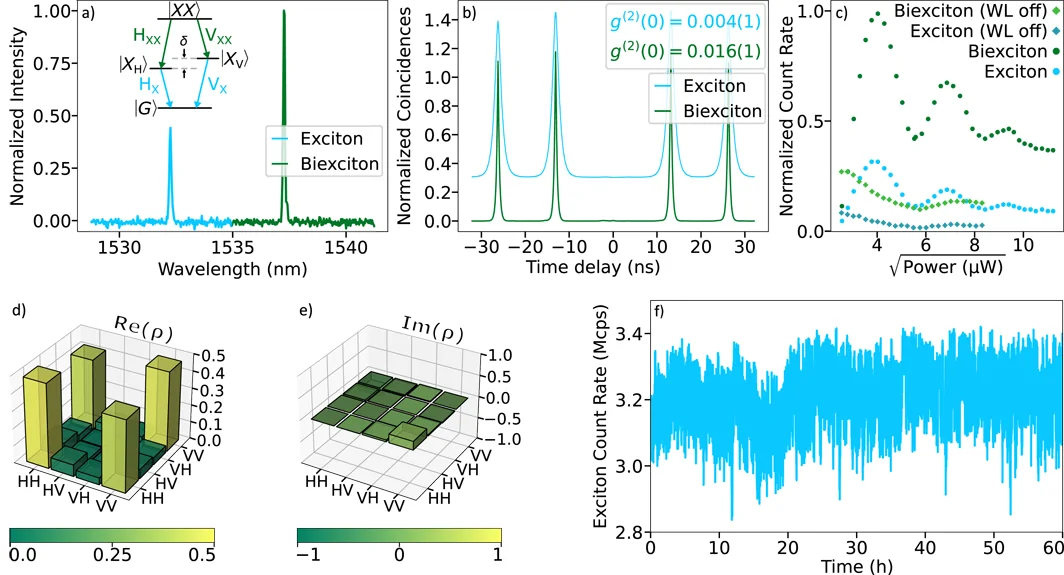
(a) Spectrum of one selected quantum dot under TPE excitation. As the laser is fully suppressed by the spectral filtering, only the QD‐TPE exciton and biexciton transitions are visible. In the inset, a schematic energy diagram of the biexciton‐exciton radiative cascade. (b) Normalised photon autocorrelation measurements, $g^{\left(2\right)}\left(\tau \right)$, for the exciton and biexciton emission, where the exciton measurement is shifted by an offset of 0.3. (c) Intensity over excitation power showing Rabi oscillations typical of coherent excitation methods for the exciton and biexciton emission. The measurements with and without the optimised white‐light illumination are presented. (d) Real part and (e) imaginary part of the reconstructed two‐photon density matrix obtained by quantum state tomography for the emitted polarisation‐entangled photon pairs, using a coincidence time window of 160 ps. Data in panels (d) and (e) are taken from Ref. [22] and reanalysed using a coincidence time window of 160 ps. (f) Long‐term measurement of the exciton count rate. The rates are averaged over a bin width of 10 ps.

After correcting for detection efficiency and the quantum tomography setup, the fibre‐coupled single‐photon count rates were measured to be 5.04 (16)Mcps for the biexciton and 1.97 (6)Mcps for the exciton at an excitation repetition rate of 76MHz as previously reported in Ref. [22]. These values correspond to end‐to‐end system efficiencies of approximately 6.6% and 2.6% for the biexciton and exciton photons, respectively. The QD‐TPE is optically active for 80.9 (3)% of the time, see the Supporting Information. The single‐photon character of both transitions was confirmed by measurements of the second‐order correlation function, yielding background‐corrected zero‐delay values of $g^{\left(2\right)}\left(0\right)=0.016\left(1\right)$ for the biexciton and $g^{\left(2\right)}\left(0\right)=0.004\left(1\right)$ for the exciton (Figure 3b). The measured Rabi oscillations further demonstrate the high fidelity of coherent state preparation and the strong influence of the two‐colour illumination. Figure 3c presents Rabi oscillations measured with and without the optimised white‐light illumination. A more detailed investigation of this effect is provided in the Supporting Information.

In addition to the high fibre‐coupled coincidence rate of 201(13) kcps [22] and the exceptionally low multi‐photon emission probabilities, QD‐TPE exhibits a fine‐structure splitting of only 3.2μ eV, providing the essential prerequisite for the generation of high‐fidelity polarisation‐entangled photon pairs. For a coincidence time window of 160 ps, an entanglement fidelity of 0.902(1) was obtained. Within this time window, the measured fibre‐coupled coincidence rate was 27.31(180) kcps, corrected for the detection efficiency of the detectors and the tomography setup. The corresponding reconstructed density matrix is shown in Figure 3d,e, while the data‐processing procedure is described in the Supporting Information.

The QD‐TPE source was also used to perform a long‐term stability measurement, during which the exciton single‐photon count rate was continuously monitored over a period of 60 h, spanning several standard working days; see Figure 3f. The rack‐integrated quantum‐dot‐based source was operated in a technical room without dedicated air conditioning, humidity control, or active vibration isolation beyond that provided by the building foundation. Over the entire measurement period, the standard deviation of the measured count rate was 2.7% for a bin width of 10 s, demonstrating the high operational stability of the source under realistic deployment conditions. For more details see the Supporting Information S1.

### 3.3 Deployed Quantum Communication

The potential of the transportable quantum‐dot‐based source of single and entangled photons for future deployed quantum communication experiments was first demonstrated by connecting the QD‐LA source to a deployed metropolitan single‐mode fibre loop. The fibre link connects the laboratory at the Institut für Halbleiteroptik und Funktionelle Grenzflächen (IHFG) on the Vaihingen campus of the University of Stuttgart with a facility of Deutsche Telekom AG in Stuttgart‐Feuerbach, where the fibre loop is turned back toward the laboratory (Figure 4a) [26]. The total fibre length is 35.8km with an overall transmission loss of 14.4dB, the majority of which originates from fibre connectors rather than fibre attenuation.

**FIGURE 4:**
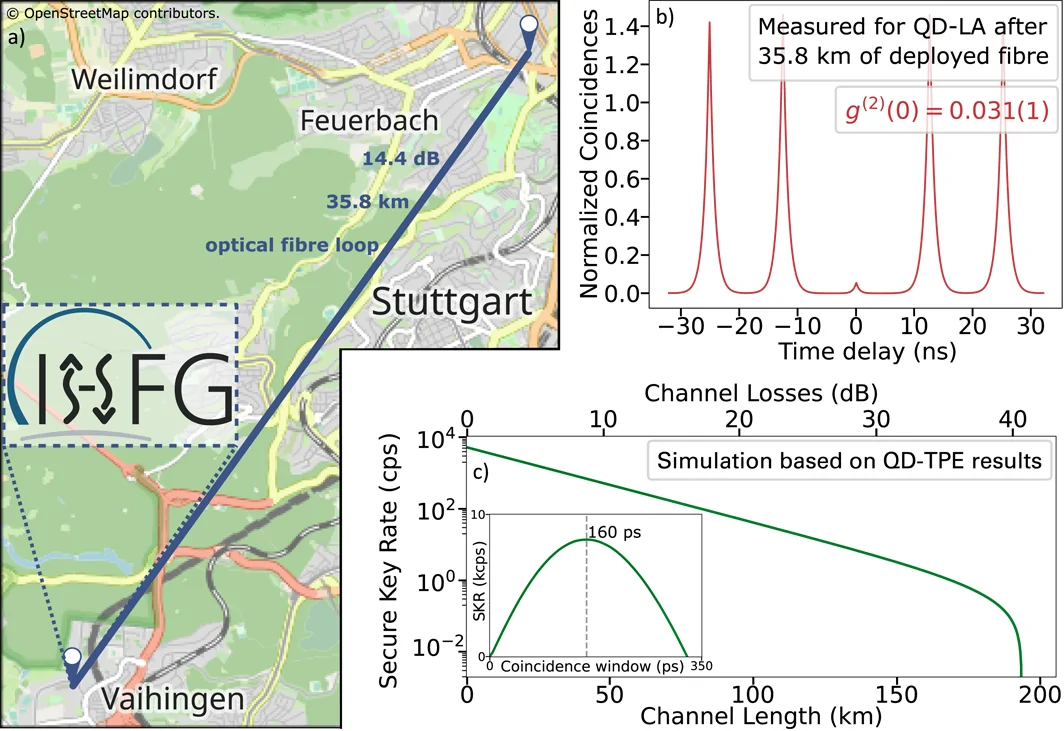
(a) Deployed metropolitan optical fibre link in Stuttgart, Germany, connecting the research laboratory of the Institut für Halbleiteroptik und Funktionelle Grenzflächen (IHFG) on the Vaihingen campus of the University of Stuttgart with the Deutsche Telekom AG site in Stuttgart‐Feuerbach. The link consists of two optical fibres connected in Stuttgart‐Feuerbach to form a fibre loop. The total loop length is 35.8km with a propagation loss of 14.4dB. The displayed map is based on OpenStreetMap data; for copyright information, see openstreetmap.org/copyright. (b) Normalised second‐order autocorrelation measurement, $g^{\left(2\right)}\left(\tau \right)$, of QD‐LA after transmission through the deployed metropolitan fibre loop. (c) Calculated secure key rate (SKR) of the BBM92 quantum key distribution protocol using QD‐TPE as a function of communication distance. The calculation assumes a single‐photon detection with an efficiency of 80% and a dark‐count probability of $10^{-6}$, an error‐correction algorithm operating at 80% efficiency and a fibre attenuation of 0.21dB/km. The inset shows the dependence of SKR on coincidence window width at zero channel length.

Single photons emitted by QD‐LA under LA phonon‐assisted excitation were transmitted through the deployed fiber loop. After correcting for the detector efficiency, a single‐photon count rate of 20 kcps was measured at the output of the link. The single‐photon purity at the saturation excitation power was quantified by the measured background‐corrected zero‐delay second‐order correlation value of $g^{\left(2\right)}\left(0\right)=$0.031 (1), as shown in Figure 4b. This value is directly comparable to the $g^{\left(2\right)}\left(0\right)=$0.063 (1) measured at saturation before transmission through the fiber loop. The small difference between the two values can be attributed to the high losses of the deployed fiber link. These results demonstrate the suitability of the source for deployed single‐photon‐based quantum communication [25, 27].

The potential of the QD‐TPE source for long‐distance entanglement‐based QKD, specifically the BBM92 protocol [28], was further investigated by calculating the secure key rate (SKR) based on the experimentally measured source and setup characteristics. The lower bound of the SKR was calculated using the measured time‐resolved density matrices, together with the experimentally determined coincidence rates and $g^{\left(2\right)}\left(0\right)$ values [29, 30]. The calculation assumes detection with an efficiency of 80% (includes single‐photon detectors efficiency and efficiency of a QKD projection measurement setup) and a dark‐count probability of $10^{-6}$, together with an error‐correction algorithm operating at 80% efficiency. A detailed description of the SKR calculation is provided in the Supporting Information S1. The coincidence time window was optimised to maximise the SKR (see Figure 4c) and was set to 160 ps, when SKR reach the maximum at zero losses.

As an example, for a communication fiber length of 150 km, corresponding to a total channel propagation loss of 31.5 dB in standard single‐mode silica optical fiber, the calculated maximum SKR reaches 3.43 cps. To the best of our knowledge, the highest SKR reported to date for an experimentally implemented 780 nm QD entangled‐photon source in a BBM92 QKD protocol is 86 cps, achieved over a 350 m‐long 780‐HP single‐mode fiber with a total communication loss of 3 dB [30, 31]. Under the same channel loss, the presented system is projected to achieve an SKR of 2850 cps, highlighting the strong potential of the presented source for future long‐distance quantum communication networks.

## 4 Conclusion

In this work, we demonstrate a compact, transportable source of single‐ and entangled‐photon pairs operating in the telecom C‐band. The setup includes all necessary elements, from the cryostat to excitation lasers and optics, for operating the telecom C‐band QD sources at any location without needing dedicated infrastructure. Thanks to the automatic control of all active elements, the user can run the experiment from a remote location. This compact optical setup enables operation of the quantum‐dot source without compromising the overall transmission efficiency of the system, which exceeds 65% after correcting for the detector efficiency. This value is indeed higher as compared to highly efficient telecom C‐band table‐top setups (with previously reported values of circa 30% [32]). Additionally, the source performances are also not impacted by operating in the rack‐based unit, as was demonstrated by the 60 hours‐long stability measurement of the QD‐TPE exciton fibre‐coupled count rate. This high setup efficiency, together with a bright source of quantum light, allowed setting a new record in the generation of polarisation‐entangled photon pairs as reported in Ref. [22]. Here, this entangled‐photon source was further investigated, demonstrating its promising potential for future long‐distance entanglement‐based QKD protocols. Based on the measured count rate and assuming realistic channel losses, the calculated maximum SKR highlights the potential of the presented system to surpass the performance of previous realisations of entanglement‐based QKD. Finally, to prove that such a setup can be integrated into existing optical fibre links, a source of single photons was interfaced with an intra‐city deployed fibre link of circa 36km without compromising the single‐photon nature of the QD emission. In conclusion, the results show the possibility of realising a high‐efficiency, compact and transportable setup operating with state‐of‐the‐art quantum dots as sources of single‐ and polarisation‐entangled photons in the telecom C‐band. This will play a major role in transforming quantum optics experiments into field‐deployed quantum technology.

## Author Contributions

**Michal Vyvlecka:** conceptualization, investigation, writing – original draft, methodology, visualization, formal analysis, data curation, supervision. **Raphael Joos:** conceptualization, investigation, validation, software, formal analysis, data curation. **Benjamin Breiholz:** investigation, writing – original draft, methodology, visualization, formal analysis, data curation, writing – review and editing. **Emma Marmasse:** investigation, methodology, software, formal analysis, data curation. **Anna Friederike Köhler:** software, data curation, formal analysis. **Ponraj Vijayan:** data curation, methodology, resources. **Tobias Huber‐Loyola:** conceptualization, funding acquisition, supervision, resources. **Sven Höfling:** resources, funding acquisition, conceptualization, supervision. **Michael Jetter:** resources, funding acquisition, supervision, conceptualization. **Simone Luca Portalupi:** writing – original draft, funding acquisition, supervision, resources, writing – review and editing, conceptualization. **Peter Michler:** conceptualization, funding acquisition, writing – original draft, supervision, resources.

## Conflicts of Interest

The authors declare no conflicts of interest.

## Supporting information


Supporting Information S1

## Data Availability

The data that support the findings of this study are available from the corresponding author upon reasonable request.
