# Supplementary material for: Rack‐Integrated Quantum Dot‐Based Source of Single and Entangled Photons at Telecom C‐Band
Source: Nanophotonics. 2026 Nov 3;15(21):e70353. doi: 10.1002/nap2.70353 (PMC13731972; doi:10.1002/nap2.70353)
Supplement: Supplementary file 1 [file NAP2-15-e70353-s001.pdf]

Supporting Information for "Rack-integrated quantum dot- based source of single and entangled photons at telecom C-band "

Michal Vyvlecka Raphael Joos Benjamin Breiholz Emma Marmasse Anna Friederike Köhler Ponraj Vijayan Tobias Huber-Loyola Sven Höfling Michael Jetter Simone Luca Portalupi Peter Michler*

Dr. Michal Vyvlecka, Raphael Joos, Benjamin Breiholz, Emma Marmasse, Anna Friederike Köhler, Ponraj Vijayan, Dr. Michael Jetter, Dr. Simone Luca Portalupi, Prof. Dr. Peter Michler
Institut für Halbleiteroptik und Funktionelle Grenzflächen, Center for Integrated Quantum Science and Technology (IQ^{ST}) and SCoPE, University of Stuttgart, Allmandring 3, 70569 Stuttgart, Germany
Email Address: michal.vyvlecka@ihfg.uni-stuttgart.de
Prof. Dr. Tobias Huber-Loyola, Prof. Dr. Sven Höfling
Julius-Maximilians-Universität Würzburg, Physikalisches Institut and Würzburg-Dresden Cluster of Excellence, ctd.qmat, Am Hubland, Würzburg, Bavaria, Germany
Prof. Dr. Tobias Huber-Loyola
Institute of Photonics and Quantum Electronics (IPQ) and Center for Integrated Quantum Science and Technology (IQST), Karlsruhe Institute of Technology (KIT), Engesserstr. 5, 76131 Karlsruhe, Germany

Keywords: *quantum dots, telecom C-band, deployed quantum communication*

SUPPORTING NOTE 1 QD-LA and QD-TPE: Blinking

To assess the optical stability of the emitters, we analyzed their blinking behavior. The blinking traces corresponding to the $g^{(2)}(\tau)$ measurements presented in the main text are shown in Fig. S1. Based on these traces, we determined the fraction of the measurement time during which the QDs were optically active- To do this, we first we fit the envelope with the model

$$C(\tau) = A \cdot \prod_{\mathrm{i}=1}^{4} \left(1 + B_{\mathrm{i}} \mathrm{e}^{-\frac{|\tau|}{T_{\mathrm{i}}}}\right), \tag{S1}$$

where the different effects, that are responsible for blinking, are represented by the amplitude B_i and the time scales T_i [1]. From these fitted parameters, we can then calculate the active time fraction of the QDs via

$$\beta = \prod_{\mathrm{i}=1}^{4} \frac{1}{1 + B_{\mathrm{i}}}. \tag{S2}$$

This gives for the QD-LA emitter an optically active time fraction of 60.9(1) %, whereas the QD-TPE emitter remained optically active for 80.9(3) % of the measurement time.

SUPPORTING NOTE 2 QD-TPE: Two-colour illumination

The performance of the QD-TPE emitter is strongly influenced by the intensity of the additional white-light illumination applied during TPE excitation. To investigate this dependence, we gradually reduced the white-light intensity and monitored the Rabi oscillations and blinking behavior. Here, 100 % corresponds to the white-light intensity at which the maximum count rate is reached, while 80 %, 60 %, and so on correspond to the intensities at which 80 %, 60 %, etc. of the maximum count rate are obtained. The Rabi oscillations of the exciton and biexciton transitions are shown in Fig. S2 a) and b), respectively. As the white-light intensity is reduced, both transitions exhibit increasingly pronounced damping, indicating a degradation of the coherent state preparation. At the same time, the reduced illumination leads to a pronounced increase in emitter blinking, as shown in Fig. S2 c).

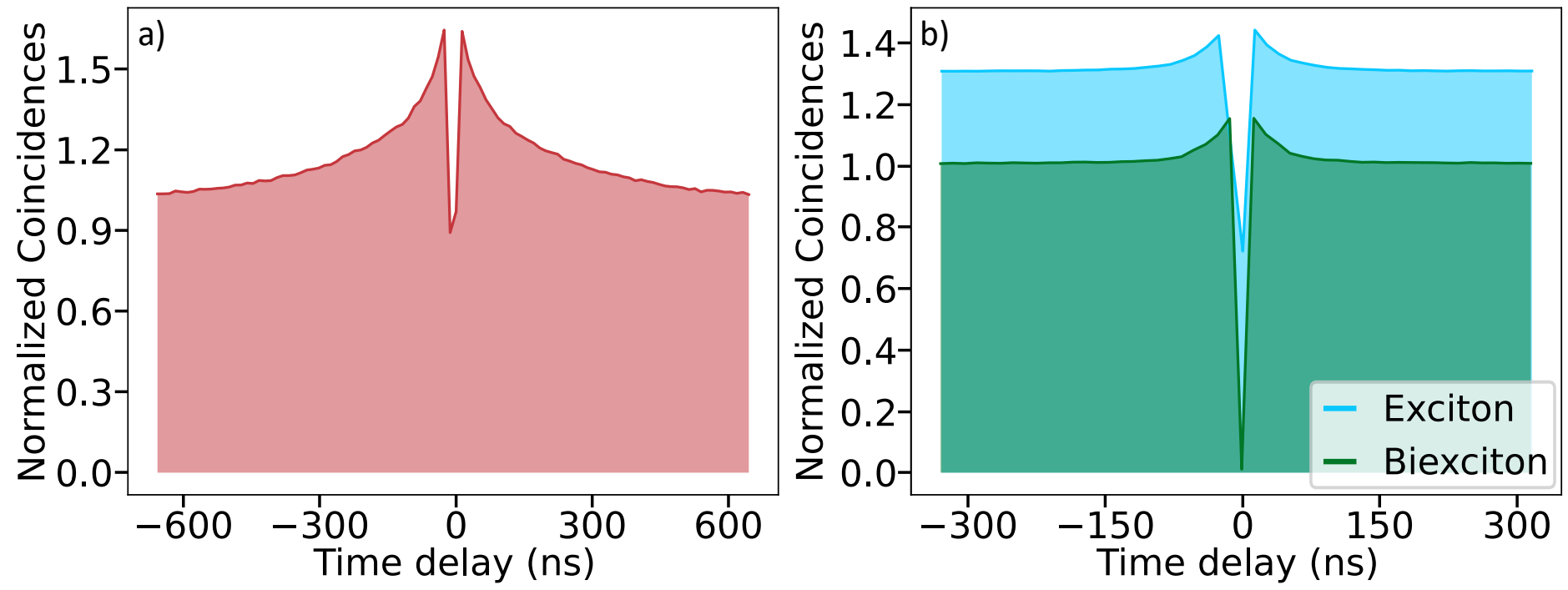

Figure S1: Blinking of the a) QD-LA and b) QD-TPE. The line of the Exciton in b) is shifted upwards by 0.3 for visual clearity.

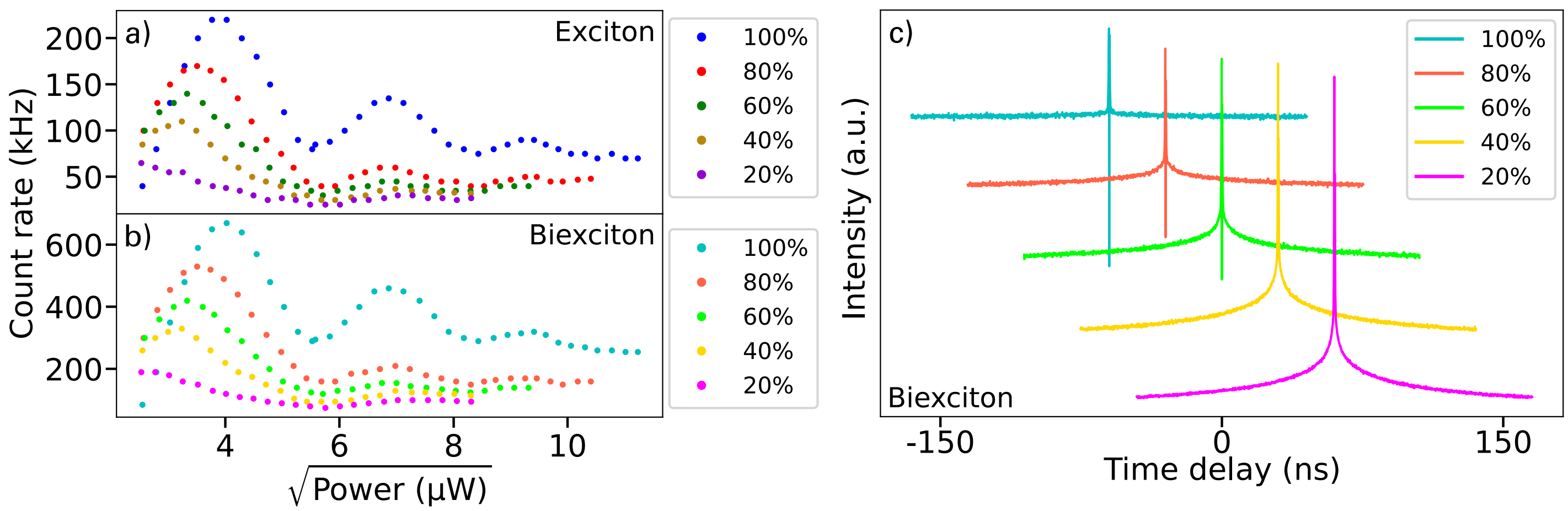

Figure S2: The Rabi oscillations depending on the white light intensity for the Exciton in a) and the Biexciton in b). c) The effect of blinking in dependence of white light intensity. Note that the blinking curves are shifted for visual clarity. 100 % resembles the white light intensity, where the maximum count rate is reached, 80 %, when 80 % of the maximum count rate is reached, and so on.

These observations demonstrate the important role of the additional white-light illumination in stabilizing the free-charge-carrier environment of the QD, leading to, not only suppressed blinking, but also enhanced brightness and fidelity of the coherent state preparation. Consequently, it significantly improves the overall performance of the single-photon source.

SUPPORTING NOTE 3 Derivation of SKR estimation with QD-TPE

The estimation follows the theoretical lower-bound expression for entanglement-based QKD after error correction and privacy amplification [2]. The SKR is given as

$$\mathrm{SKR} \geq \frac{1}{2}\left[Q_1(1 - H_2(\epsilon_1)) - f Q_\mu H_2(E_\mu)\right] \ , \tag{S3}$$

where Q_μ denotes the total gain of the signal state, E_μ is the total error rate, Q_1 the single-photon gain, ϵ_1 the single-photon error rate, f the error-correction inefficiency assuming one-way classical error correction, and $H_2(x)$ the binary Shannon entropy. These variables are defined as follows:

$$Q_\mu = \sum_{\mathrm{k}=0}^{\infty} Q_{\mathrm{k}}, \tag{S4}$$

$$Q_{\mathrm{k}} = P_\mu(\mathrm{k}) Y_{\mathrm{k}}, \tag{S5}$$

$$E_\mu = \frac{1}{Q_\mu} \sum_{\mathrm{k}=0}^{\infty} \epsilon_{\mathrm{k}} Q_{\mathrm{k}}, \tag{S6}$$

$$\epsilon_{\mathrm{k}} = \frac{\epsilon_0 Y_0 + \mathrm{QBER}\left[1-(1-\eta_{\mathrm{d}}\eta_{\mathrm{t}})^{\mathrm{k}}\right]}{Y_{\mathrm{k}}}\ , \tag{S7}$$

$$Y_{\mathrm{k}} = Y_0 + (1-Y_0)\left[1-(1-\eta_{\mathrm{d}}\eta_{\mathrm{t}})^{\mathrm{k}}\right], \tag{S8}$$

where η_{d} is Bob's detection efficiency, ηt the channel transmission, Y_0 the dark-count probability, Y_{k} is the k-photon yield, QBER is quantum bit error rate, and $\epsilon_0 = 1/2$ corresponds to random errors originating from detector dark counts. The $P_\mu(\mathrm{k})$ corresponds to the probability of emission of k photons. Since TPE-excited quantum dots exhibit an extremely low multi-photon contribution, we assume $P_\mu(\mathrm{k} \geq 3) = 0$ [3]. The remaining two-photon probability is conservatively bounded using the experimentally measured source brightness B and the second-order correlation function $g^{(2)}(0)$ [4] with

$$P_\mu(2) \leq \frac{1 - Bg^{(2)}(0) - \sqrt{1-2Bg^{(2)}(0)}}{g^{(2)}(0)}\ . \tag{S9}$$

Then the probabilities of emitting 0 and 1 photon can be defined as

$$P_\mu(0) = 1 - B\ , \tag{S10}$$

$$P_\mu(1) = B - P_\mu(2)\ . \tag{S11}$$

The QBER is estimated from the experimentally measured density matrix of the polarization-entangled $\hat{\rho}$ state [5] as

$$QBER = \frac{1}{2}\sum_{\mathrm{j}=1}^{4} \langle O_{\mathrm{j}}|\, \hat{\rho}\, |O_{\mathrm{j}}\rangle\ , \tag{S12}$$

where $|O_{\mathrm{j}}\rangle \in \{|H\rangle_{\mathrm{A}}|V\rangle_{\mathrm{B}}, |V\rangle_{\mathrm{A}}|H\rangle_{\mathrm{B}}, |+\rangle_{\mathrm{A}}|-\rangle_{\mathrm{B}}, |-\rangle_{\mathrm{A}}|+\rangle_{\mathrm{B}}\}$ corresponds to cross-correlation measurements between Alice's and Bob's photons in the conjugate basis. Finally, with all this, we can then compute the SKR with the help of S3, which is shown in Fig. 4 c).

In the SKR calculation, we assume a single-photon detection efficiency of $\eta_d = 80\,\%$ and a dark-count probability of $Y_0 = 10^{-6}$. Here, η_d also includes the transmission of the 50:50 beamsplitter required for the random choice of measurement basis in a BBM92 QKD implementation. This beamsplitter is not currently present in our measurement setup. The assumed dark-count probability represents a conservative upper bound for the corresponding coincidence time window. The error-correction efficiency was realistically set to 80 %, corresponding to $f = 1.2$. It is important to note here that this is a rather conservative assumption of the error code efficiency. The remaining experimental imperfections, including polarization drift from the laboratory setup and other errors coming from the setup imperfections, are included in the experimentally reconstructed density matrix $\hat{\rho}$ and therefore enter the QBER used in the SKR calculation. The polarisation drift induced by the deployed fibre link is not considered in the calculation. Although previous measurements on a 36 km deployed fiber link showed minimal polarization drift for measurement times below one hour [6], longer measurement times will require active polarization stabilization. Finally, we have analyzed the dependence of the projected SKR on the coincidence time window. The optimal coincidence window was chosen as 160 ps, when SKR reach the maximum at zero losses.

SUPPORTING NOTE 4 Density-matrix reconstruction

In the following, we provide details on the reconstruction of the density matrix. The density matrix was reconstructed using a maximum-likelihood procedure, while the uncertainties were determined through a Monte Carlo analysis based on the measured counting statistics.

The parameters used for the reconstruction and statistical analysis are as follows. The combined timing jitter of the detectors and time tagger was 80 ps, and the histogram bin width was 160 ps. The average number of recorded events per measurement basis setting was 272 801 counts, corresponding to an integration time of 400 s per basis setting.

SUPPORTING NOTE 5 Long-term stability

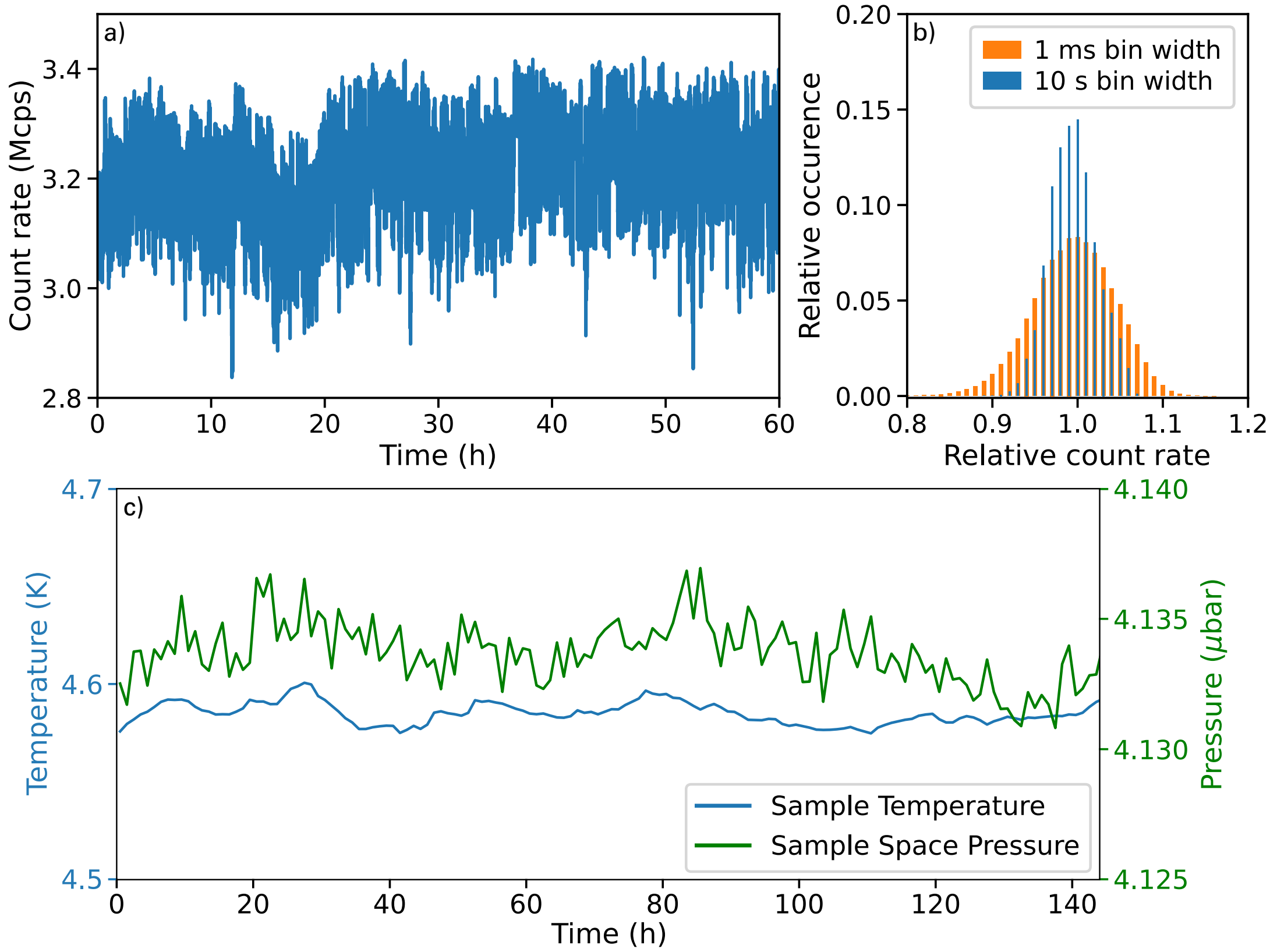

Figure S3: a) Long-term measurement of the count rate. The rates are averaged over a bin width of 10 s. b) Comparison of the occurrence of different count rates between two timescales. One being at shorter timescales, where the rates were averaged over a bin width of 1 ms, and on longer timescales, averaged over 10 s. c) Long-term measurement of sample temperature and sample space pressure.

Stability is a crucial factor for structures intended for deployment and transport. To assess the stability of our system under realistic operating conditions, all measurements were performed with the rack system placed inside its transportation box in a technical room. The room is neither air-conditioned nor humidity-controlled and provides no dedicated vibration damping beyond the foundation of the building. Thus, the measurements were carried out under conditions that closely resemble those expected during deployment outside a controlled laboratory environment.

To evaluate the long-term stability, we monitored the count rate continuously for 60 h. The resulting time trace is shown in Fig. S3 a). Over the entire measurement period, the count rate remains stable at approximately 3.2 Mcps. For this plot, the count rate was averaged over a bin width of 10 s in order to enable illustration of the full range.

To further investigate the stability on different timescales, Fig. S3 b) shows the relative occurrence of the measured count rates for bin widths of 10 s and 1 ms. On the shorter timescale, the count-rate distribution is broader, primarily due to short-timescale fluctuations, such as those associated with the operation of the cold-head. On longer timescales, the distribution becomes narrower, indicating stable operation over extended periods. Importantly, neither distribution shows any major fluctuations over several days of continuous operation, demonstrating the long-term stability of the system. The corresponding temperature and pressure variations are shown in Fig. S3 c).

Despite these fluctuations, the average count rate remains essentially unchanged across the two timescales. The corresponding values obtained from the distributions in Fig. S3 b) are 3.18(9) Mcps for a bin width of 10 s and 3.18(15) Mcps for 1 ms.

References

[1] J.-P. Jahn, M. Munsch, L. Béguin, A. V. Kuhlmann, M. Renggli, Y. Huo, F. Ding, R. Trotta, M. Reindl, O. G. Schmidt, A. Rastelli, P. Treutlein, R. J. Warburton, *Phys. Rev. B* **2015**, *92* 245439.

[2] X. Ma, Quantum cryptography: theory and practice, **2008**, URL https://arxiv.org/abs/0808.1385.

[3] L. Hanschke, K. A. Fischer, S. Appel, D. Lukin, J. Wierzbowski, S. Sun, R. Trivedi, J. Vučković, J. J. Finley, K. Müller, *npj Quantum Information* **2018**, *4*, 1 43.

[4] M. Vyvlecka, L. Jehle, C. Nawrath, F. Giorgino, M. Bozzio, R. Sittig, M. Jetter, S. L. Portalupi, P. Michler, P. Walther, *Applied Physics Letters* **2023**, *123*, 17 174001.

[5] C. Schimpf, M. Reindl, D. Huber, B. Lehner, S. F. Covre Da Silva, S. Manna, M. Vyvlecka, P. Walther, A. Rastelli, *Science Advances* **2021**, *7*, 16.

[6] T. Strobel, S. Kazmaier, T. Bauer, M. Schäfer, A. Choudhary, N. Lal Sharma, R. Joos, C. Nawrath, J. H. Weber, W. Nie, et al., *Optica Quantum* **2024**, *2*, 4 274.
